# Serum antioxidant vitamins and respiratory morbidity and mortality: a pooled analysis

**DOI:** 10.1186/s12931-022-02059-w

**Published:** 2022-06-09

**Authors:** Paivi M. Salo, Angelico Mendy, Jesse Wilkerson, Samantha A. Molsberry, Lydia Feinstein, Stephanie J. London, Michael B. Fessler, Peter S. Thorne, Darryl C. Zeldin

**Affiliations:** 1 Division of Intramural Research, National Institute of Environmental Health Sciences, National Institutes of Health, Research Triangle Park, NC USA; 2 Division of Epidemiology, Department of Environmental and Public Health Sciences, University of Cincinnati College of Medicine, Cincinnati, OH USA; 3 Social & Scientific Systems, Durham, NC USA; 4 Department of Occupational and Environmental Health, University of Iowa, IA, USA

**Keywords:** Antioxidant, Morbidity, Mortality, Respiratory disease, Serum vitamin

## Abstract

**Background:**

Oxidative stress plays a key role in the pathogenesis of respiratory diseases; however, studies on antioxidant vitamins and respiratory outcomes have been conflicting. We evaluated whether lower serum levels of vitamins A, C, D, and E are associated with respiratory morbidity and mortality in the U.S. adult population.

**Methods:**

We conducted a pooled analysis of data from the 1988–1994 and 1999–2006 National Health and Nutrition Examination Survey (participants aged ≥ 20 years). We estimated covariate-adjusted odds ratios (aOR) per interquartile decrease in each serum vitamin level to quantify associations with respiratory morbidity, and covariate-adjusted hazard ratios (aHR) to quantify associations with respiratory mortality assessed prospectively through 2015. Vitamin supplementation and smoking were evaluated as potential effect modifiers.

**Results:**

Lower serum vitamin C increased the odds of wheeze among all participants (overall aOR: 1.08, 95% CI: 1.01–1.16). Among smokers, lower serum α-tocopherol vitamin E increased the odds of wheeze (aOR: 1.11, 95% CI: 1.04–1.19) and chronic bronchitis/emphysema (aOR: 1.13, 95% CI: 1.03–1.24). Conversely, lower serum γ-tocopherol vitamin E was associated with lower odds of wheeze and chronic bronchitis/emphysema (overall aORs: 0.85, 95% CI: 0.79–0.92 and 0.85, 95% CI: 0.76–0.95, respectively). Lower serum vitamin C was associated with increased chronic lower respiratory disease (CLRD) mortality in all participants (overall aHR: 1.27, 95% CI: 1.07–1.51), whereas lower serum 25-hydroxyvitamin D (25-OHD) tended to increase mortality from CLRD and influenza/pneumonia among smokers (aHR range: 1.33–1.75). Mortality from influenza/ pneumonia increased with decreasing serum vitamin A levels in all participants (overall aHR: 1.21, 95% CI: 0.99–1.48). In pooled analysis, vitamin C deficiency and 25-OHD insufficiency were associated with mortality from influenza/pneumonia, increasing mortality risk up to twofold.

**Conclusions:**

Our analysis of nationally representative data on over 34,000 participants showed that lower serum levels of vitamins A, C, D, and α-tocopherol vitamin E are associated with increased respiratory morbidity and/or mortality in U.S. adults. The results underscore the importance of antioxidant vitamins in respiratory health.

**Supplementary Information:**

The online version contains supplementary material available at 10.1186/s12931-022-02059-w.

## Background

Oxidative stress is characterized by an imbalance between production of free radicals and antioxidant defenses [1, 2]. Free radicals are highly reactive molecules with unpaired electrons that can trigger cellular and molecular processes leading to cell and tissue damage. They include reactive oxygen species (ROS) and reactive nitrogen species (RNS) [1]. ROS are generated from environmental pollutants, mitochondrial respiration, and phagocytic oxidative burst, whereas RNS result from the interaction between ROS and nitrite [1–3]. Enzymatic and nonenzymatic antioxidants counteract oxidative stress by stabilizing, scavenging, and suppressing the production of oxidants and free radicals [1–3]. Vitamins A (retinols), C (ascorbic acid) and E (α-tocopherol) are major nonenzymatic antioxidants, but vitamin D (calciferol) has also been shown to have antioxidant properties [2–5]. In addition to neutralizing free radicals, vitamin A and α-tocopherol reduce lipid peroxidation, while vitamin C, which is abundant in tissues, deactivates ROS in fluids and regenerates cellular vitamin E from its oxidized form [2]. γ-tocopherol, another isoform of vitamin E, has a similar capacity to scavenge ROS during peroxidation, but can additionally react with RNS [6]. Yet, recent reports suggest that the two isoforms of vitamin E might have differential effects on regulation of inflammation [6, 7]. Vitamin D can reduce lipid peroxidation and act through the vitamin D receptor to reduce free radical production [5, 8]. The primary source of vitamins A, C, and E precursors is diet; sun exposure is the primary source of vitamin D, but it can also be obtained through dietary intake [9, 10].

Lungs are particularly vulnerable to the effects of oxidative stress because of their exposure to high levels of oxygen, large surface area, and abundant vascularization. Furthermore, inhalation of toxicants or pathogens and activation of resident or inflammatory cells in response to injury can contribute to the depletion of antioxidant capacities [11]. Oxidative stress is well-documented to play a role in the pathogenesis of respiratory diseases. In asthma, it promotes T-helper type 2 responses and increases ROS in antigen-presenting cells, leading to allergic airway inflammation [12]. It also enhances bronchoconstriction, mucin secretion, and chemokine production to cause asthma exacerbations [12]. In chronic obstructive pulmonary disease (COPD), ROS and carbonyls are associated with airway inflammation and remodeling and can cause corticosteroid resistance [13]. Moreover, oxidative stress can enhance cell injury and increase disease severity in influenza A infection, which is a major cause of pneumonia-related death [14].

Several studies have investigated the role of antioxidant vitamins in the pathogenesis of respiratory disease, but findings have been inconsistent [9, 15–17]. Many of these studies have relied on self-reported dietary intake of antioxidant vitamins, rather than objectively measured serum vitamin levels [9, 18], which has been shown to bias estimates due to incomplete or inaccurate reporting of food or beverage intake [19]. Despite the increasing interest in studying the role of serum antioxidant vitamins in respiratory health [15, 17], population-based studies examining the relationship between serum vitamin levels and respiratory mortality are limited [20, 21]. Our comprehensive, nationally representative pooled analysis of data from the National Health and Nutrition Examination Survey (NHANES) III and continuous NHANES is the largest study, including data from over 34,000 participants, to investigate whether levels of serum antioxidant vitamins A, C, D, and E are associated with respiratory morbidity and mortality among adults in the United States.

## Methods

### Data source

We used data from the National Health and Nutrition Examination Survey (NHANES) conducted from 1988 to 1994 (NHANES III) and from 1999 to 2006 (continuous NHANES), including all survey cycles with data on serum antioxidant vitamins A, C, D in the form of 25-hydroxyvitamin D (25-OHD), and E (α- and γ-tocopherol isoforms) (Additional file 1: Fig. S1). The NHANES is a survey of the non‐institutionalized civilian population conducted by the National Center for Health Statistics (NCHS) of the Centers for Disease Control and Prevention (CDC). It uses a complex multistage sampling design to obtain nationally representative samples of the U.S. population [22]. Data on at least one serum antioxidant vitamin were available for 34,056 adult participants aged 20 years and older (16,218 in NHANES III, and 17,838 in continuous NHANES). Data on serum vitamin A and the α-tocopherol isoform of vitamin E were available for 15,965 participants in NHANES III and for 17,733 and 17,139 participants (respectively) in continuous NHANES (1999–2006), while data on serum vitamin C were available for 15,185 participants in NHANES III and for 8,892 participants in continuous NHANES (2003–2006). Data on serum 25-OHD were available for 15,928 participants in NHANES III and 13,640 participants in continuous NHANES (2001–2006), whereas data on the γ-tocopherol isoform of vitamin E were available only for continuous survey cycles 1999–2006 (N = 17,139). All NHANES protocols were approved by the NCHS and the CDC’s Institutional Review Boards and informed consent was obtained from all participants.

### Serum antioxidant vitamins

Serum concentrations of vitamins A, C, and E (α- and γ-tocopherol) were measured using isocratic high-performance liquid chromatography [23–25]. Because the analytic methods used to measure serum vitamin C differed between NHANES III and the continuous NHANES, NHANES III vitamin C values were adjusted to match the methods used by the continuous NHANES, using the formula 1.0566*(original NHANES III)—1.9345 μmol/L. Serum 25-OHD levels were measured using the DiaSorin assay which extracted hydroxylated metabolites from serum and assayed the samples using an antibody specific to 25-OHD. Due to radioimmunoassay reformulation between the survey cycles, all 25-OHD values were standardized and expressed in LC–MS/MS equivalents [26, 27]. Details on the laboratory procedures are provided elsewhere (https://www.cdc.gov/nchs/data/nhanes/nhanes_05_06/vid_d.pdf). Vitamin A, C, and E deficiency was defined using clinically established cut-points of serum levels < 20.1 µg/dl for vitamin A, < 0.20 mg/dl for vitamin C, and < 0.50 mg/dl for vitamin E based on α-tocopherol levels [28–30]. Serum 25-OHD was defined as insufficient at levels < 20 ng/mL or deficient at < 12 ng/mL, as suggested by the literature [31, 32].

### Respiratory morbidity and mortality

Current asthma, wheeze in the past 12 months, and chronic bronchitis or emphysema were assessed via self-report at baseline using questionnaires. Participants had current asthma if their asthma was physician-diagnosed and still affecting them at the time of the survey. Participants had chronic bronchitis or emphysema if they reported a physician-diagnosis of the condition or, to mitigate concerns of underdiagnosis, if they met our clinical definition of chronic bronchitis (cough and phlegm for at least three months in the year and for at least two years). Mortality from CLRD, influenza, or pneumonia from enrollment through 2015 was ascertained via linkage to the National Death Index and death certificates [33]. The causes of death were identified using the 10th Revision of the International Classification of Diseases (ICD‐10) codes. Deaths occurring before 1999 were originally coded with the previous version of the classification (i.e., ICD-9 codes) but were recoded into comparable ICD-10 codes by the NCHS. Chronic lower respiratory disease (CLRD) mortality was defined as death from asthma (J45-J46), emphysema (J43), bronchitis (chronic or other; J40-J42), or other CLRD (J44 and J47). Mortality from influenza or pneumonia was defined as death from influenza (J9-J11) or pneumonia (J12-J18).

### Covariates

Baseline information on age, sex, race/ethnicity, annual household income, educational attainment, cigarette smoking and exposure to cigarette smoke, pack-years of cigarette smoking, alcohol consumption, caloric intake, general health status, and current use of any vitamin supplements in the past month were self-reported via questionnaire. Alcohol consumption was categorized based on average weekly alcohol consumption as follows: heavy drinker (male: ≥ 14 drinks/week; female ≥ 7 drinks/week), light/moderate drinker (male: 1–13 drinks/week; female: 1–6 drinks/week), and non-drinker (0 drinks/week). Caloric intake was measured using a 24 h diet recall and categorized into tertiles of total energy consumption (< 1,638 kcal, 1,638–2,477 kcal, > 2,477 kcal in NHANES III; < 1,670 kcal, 1,670–2,475 kcal, > 2,475 kcal in continuous NHANES). Self-perceived general health status was assessed on an ordinal scale using the following categories: excellent or very good, good, and fair or poor. Body mass index (BMI) was calculated as weight (in kg) divided by height (in m^2^) and was categorized as underweight (< 18.5 kg/m^2^), normal (18.5–24.9 kg/m^2^), overweight (25–29.9 kg/m^2^), and obese (≥ 30 kg/m^2^). Season of blood collection was categorized as either April-September or October–March, based on the participant’s date of blood collection. Total serum cholesterol was measured enzymatically in reactions hydrolyzing cholesteryl esters and oxidizing the hydroxyl group of cholesterol [34].

### Statistical analysis

Descriptive statistics were used to summarize the serum vitamin levels overall and by study participants’ characteristics, using the Kruskal–Wallis H and Dwass-Steel-Critchlow-Flinger tests to assess statistical differences between groups. Serum levels of vitamins A and E were log_10_-transformed to improve normality, whereas the distribution of serum vitamin C and 25-OHD levels approached normality and did not require transformation. Logistic regression was used to estimate the covariate-adjusted odds ratios (aOR) and 95% confidence intervals (CI) for the cross-sectional associations between serum vitamins and respiratory morbidity. Cox proportional hazards regression was used to estimate covariate-adjusted cause-specific hazard ratios (aHR) and 95% CIs for the associations between serum vitamin levels and respiratory mortality. In these Cox models, follow-up began with time 0 at study enrollment and continued until the event of interest (death due to CLRD or influenza/pneumonia) or a censoring event (death from another cause or December 31, 2015), whichever occurred first; due to the cross-sectional nature of NHANES data, covariates were captured only at enrollment, and thus were time invariant in these models. The proportional hazards assumption was assessed using Schoenfeld residuals and no violations were identified. Odds and hazard ratios were scaled to report a 30-percent decrease in vitamins A and E, corresponding to an interquartile range decrease in the log_10_-transformed concentrations, while a 0.65 mg/dl decrease in vitamin C and a 12 ng/mL decrease in 25-OHD, corresponded to respective interquartile range decreases in the absolute vitamin levels. Because of differences in the complex survey designs and potential secular changes over time between NHANES III and the continuous NHANES, these datasets were not directly combined. Rather, we estimated all models separately for each NHANES cohort and used an inverse variance weighted random-effects meta-analysis approach to pool the cohort-specific effect estimates [35] and obtain overall estimates. When pooling the effect estimates, tests of heterogeneity [36, 37] were conducted to evaluate potential differences between the two cohorts. All models were adjusted for age at enrollment (continuous), gender, race/ethnicity, income, educational attainment, use of any vitamin supplements in the previous month, cigarette smoking and exposure to cigarette smoke, pack-years of cigarette smoking, BMI, alcohol consumption, caloric intake, general health status, and 2-year survey cycle (continuous NHANES only; NHANES III was a single survey cycle). Models for serum 25-OHD were further adjusted for season of blood collection, and vitamin E models were additionally adjusted for total serum cholesterol.

To evaluate potential effect modification, we conducted subgroup analyses to determine whether the associations between serum antioxidant vitamins and respiratory morbidity and mortality were modified by supplemental vitamin use (yes vs no) or smoking status (ever smoker vs never smoker), factors that can influence serum antioxidant levels directly or indirectly [38]. We assessed the potential for effect modification by first estimating models with an interaction term between each exposure variable (i.e., serum vitamins A, C, D, or E) and effect modifier of interest (i.e., supplement use or smoking status) and then by running models stratified by levels of the effect modifiers. The results of these models are provided in Additional file 1: Table S1 (interaction term *P*-values) and 2–3 (stratum-specific associations for modification by supplement use and smoking status, respectively). We emphasize the stratified results in the text only when the interaction term was significant (*P*_interaction_ < 0.05).

All analyses were performed using SAS Version 9.4 (SAS Institute, Cary, NC), accounting for the NHANES sampling weights and complex survey design to provide nationally representative estimates for the U.S. adult population. Two-sided *P*-values < 0.05 were considered statistically significant in all analyses, including analyses to assess interaction terms.

## Results

In NHANES III, 5.1% of participants had current asthma, 17.4% had wheeze in the past 12 months, and 7.8% had chronic bronchitis/emphysema (Table 1). In the continuous NHANES, the outcome prevalences were similar (6.1%, 15.5%, and 6.9%, respectively; Table 2). Among the participants included in the study, the median follow-up for mortality was 23.0 years in NHANES III and 12.3 years in the continuous NHANES. During follow-up, 459 participants died of CLRD (NHANES III: 267; continuous NHANES: 192) and 266 died of influenza/pneumonia (NHANES III: 182; continuous NHANES: 84). The levels of serum vitamins overall and by participant characteristics are shown in Table 1 (NHANES III) and Table 2 (continuous NHANES). The prevalence of vitamin A deficiency was 0.2% in both cohorts, the prevalence of vitamin C deficiency was 15.1% in NHANES III and 7.0% in the continuous NHANES, and the prevalence of 25-OHD deficiency was 6.6% in NHANES III and 6.1% in the continuous NHANES. The prevalence of 25-OHD insufficiency was 31.8% in NHANES III and 30.7% in the continuous NHANES. No participants with vitamin E deficiency were identified in either cohorts.

**Table 1:** Concentrations of vitamins A, C, D, and E (α-tocopherol) by characteristics of study participants, NHANES III (1988–1994)

	% participants	Vitamin A (µg/dl)		Vitamin C (mg/dl)		25-OHD (ng/mL)		Vitamin E [α-Tocopherol (mg/dl)]	
Characteristics		Median (Q1,Q3)	*P*	Median (Q1,Q3)	*P*	Median (Q1,Q3)	*P*	Median (Q1,Q3)	*P*
All participants	100	56.6 (47.1, 67.5)		0.74 (0.36, 1.04)		24.4 (18.2, 31.2)		1.04 (0.85, 1.31)	
Age groups									
20–39 years	46.5	53.5 (44.5, 63.7)	Ref.	0.70 (0.31, 0.99)	Ref.	25.7 (19.0, 32.8)	Ref.	0.91 (0.78, 1.11)	Ref.
40–59 years	31.8	57.5 (48.3, 68.8)	< 0.001	0.74 (0.35, 1.03)	0.992	23.7 (17.8, 30.0)	0.003	1.13 (0.93, 1.40)	< 0.001
60 + years	21.6	61.8 (52.1, 73.3)	< 0.001	0.87 (0.51, 1.18)	< 0.001	23.1 (17.4, 29.0)	0.022	1.27 (1.03, 1.62)	< 0.001
Sex									
Men	47.8	60.5 (51.7, 70.7)	< 0.001	0.67 (0.29, 0.96)	< 0.001	25.8 (20.0, 32.4)	< 0.001	1.04 (0.85, 1.28)	< 0.001
Women	52.2	52.3 (43.3, 63.6)		0.82 (0.44, 1.13)		23.0 (16.7, 29.8)		1.05 (0.86, 1.34)	
Race/ethnicity									
Non-Hispanic Whites	76.4	58.0 (48.6, 68.7)	Ref.	0.77 (0.36, 1.08)	Ref.	26.3 (20.4, 32.8)	Ref.	1.08 (0.87, 1.36)	Ref.
Non-Hispanic Blacks	10.7	51.2 (42.1, 62.4)	< 0.001	0.57 (0.25, 0.87)	< 0.001	15.6 (11.9, 20.6)	< 0.001	0.91 (0.76, 1.11)	< 0.001
Mexican Americans	5.3	51.7 (43.0, 61.1)	< 0.001	0.70 (0.38, 0.96)	< 0.001	20.8 (16.0, 26.3)	< 0.001	1.00 (0.83, 1.23)	< 0.001
Other	7.6	51.8 (43.3, 64.1)	< 0.001	0.76 (0.47, 1.00)	0.582	20.4 (15.8, 26.8)	< 0.001	0.98 (0.82, 1.25)	< 0.001
Income									
< $20,000	33.2	54.9 (45.5, 66.6)	< 0.001	0.63 (0.25, 0.98)	< 0.001	23.1 (16.8, 29.7)	< 0.001	0.99 (0.81, 1.26)	< 0.001
$20,000 +	66.8	57.4 (47.9, 67.9)		0.78 (0.43, 1.07)		25.1 (19.0, 32.1)		1.07 (0.87, 1.34)	
Education levels									
Below high school graduate	24.9	56.3 (46.9, 67.2)	< 0.001	0.61 (0.23, 0.97)	< 0.001	23.3 (17.4, 29.6)	< 0.001	1.02 (0.83, 1.31)	< 0.001
High school graduate/Some college	54.5	55.9 (46.3, 66.4)	< 0.001	0.73 (0.35, 1.04)	< 0.001	24.3 (17.9, 31.4)	< 0.001	1.03 (0.84, 1.29)	< 0.001
College graduate or higher	20.6	58.5 (49.4, 69.6)	Ref.	0.86 (0.60, 1.11)	Ref.	25.9 (20.3, 32.5)	Ref.	1.10 (0.90, 1.36)	Ref.
Cigarette smoke exposure									
No smoke exposure	8.5	57.2 (45.9, 68.0)	Ref.	0.95 (0.71, 1.22)	Ref.	24.1 (18.0, 30.8)	Ref.	1.17 (0.94, 1.52)	Ref.
Secondhand smoke exposure	37.3	55.1 (45.4, 65.6)	< 0.001	0.81 (0.48, 1.07)	< 0.001	24.1 (18.0, 30.4)	< 0.001	1.02 (0.84, 1.28)	< 0.001
Past smokers (Quit > 5 years ago)	18.4	60.9 (51.9, 72.5)	< 0.001	0.85 (0.53, 1.10)	< 0.001	25.4 (19.7, 31.6)	< 0.001	1.18 (0.96, 1.50)	1.000
Past smokers (Quit within 5 years)	7.1	58.8 (49.2, 69.6)	0.360	0.75 (0.40, 1.01)	< 0.001	24.4 (18.2, 32.0)	1.000	1.06 (0.85, 1.30)	< 0.001
Current smokers	28.6	54.9 (46.3, 65.6)	0.057	0.43 (0.15, 0.84)	< 0.001	24.1 (17.8, 32.0)	< 0.001	0.96 (0.80, 1.19)	< 0.001
BMI									
Underweight	2.4	46.3 (39.9, 56.5)	< 0.001	0.72 (0.30, 1.13)	0.22	24.5 (17.8, 32.8)	0.295	0.91 (0.79, 1.10)	0.001
Normal	42.3	54.6 (45.4, 65.5)	Ref.	0.80 (0.41, 1.11)	Ref.	26.0 (19.6, 33.0)	Ref.	0.99 (0.81, 1.24)	Ref.
Overweight	32.9	59.2 (49.7, 69.7)	< 0.001	0.74 (0.36, 1.02)	< 0.001	24.5 (18.5, 31.0)	< 0.001	1.11 (0.90, 1.41)	< 0.001
Obese	22.4	56.9 (47.2, 68.0)	0.994	0.63 (0.30, 0.95)	< 0.001	21.4 (16.0, 27.4)	< 0.001	1.08 (0.89, 1.33)	< 0.001
Alcohol consumption									
Heavy drinker	10.3	61.9 (51.0, 73.4)	< 0.001	0.61 (0.21, 0.97)	< 0.001	25.0 (18.6, 32.8)	0.016	1.01 (0.84, 1.26)	< 0.001
Light/moderate drinker	44.3	57.6 (48.9, 67.7)	< 0.001	0.74 (0.39, 1.02)	< 0.001	26.0 (19.7, 32.6)	< 0.001	1.03 (0.84, 1.26)	< 0.001
Non-drinker	45.4	54.0 (44.5, 65.0)	Ref.	0.77 (0.38, 1.08)	Ref.	23.0 (17.1, 29.5)	Ref.	1.07 (0.86, 1.37)	Ref.
Taking vitamin supplements									
Yes	42.2	58.8 (48.7, 70.1)	< 0.001	0.93 (0.63, 1.20)	< 0.001	25.5 (19.5, 32.1)	< 0.001	1.20 (0.96, 1.54)	< 0.001
No	57.8	54.9 (45.8, 65.6)		0.58 (0.24, 0.91)		23.5 (17.4, 30.5)		0.96 (0.80, 1.17)	
Caloric intake tertiles									
3rd (higher)	33.3	58.8 (49.0, 68.9)	< 0.001	0.69 (0.32, 0.98)	< 0.001	26.2 (20.0, 32.8)	< 0.001	1.02 (0.83, 1.24)	< 0.001
2nd	34.0	56.5 (47.0, 67.0)	0.279	0.76 (0.37, 1.07)	0.817	24.8 (18.6, 31.6)	< 0.001	1.04 (0.85, 1.33)	< 0.001
1st (lower)	32.6	54.4 (44.8, 65.8)	Ref.	0.78 (0.40, 1.11)	Ref.	22.3 (16.8, 29.1)	Ref.	1.07 (0.87, 1.38)	Ref.
General health status									
Excellent or very good	51.3	56.6 (47.4, 67.3)	Ref.	0.80 (0.45, 1.07)	Ref.	25.9 (19.8, 32.3)	Ref.	1.04 (0.86, 1.30)	Ref.
Good	32.9	56.7 (46.6, 67.3)	< 0.001	0.69 (0.30, 1.03)	< 0.001	23.5 (17.4, 30.7)	< 0.001	1.04 (0.83, 1.31)	0.061
Fair or poor	15.8	56.4 (46.7, 68.3)	0.728	0.63 (0.25, 0.98)	< 0.001	21.4 (16.1, 27.9)	< 0.001	1.08 (0.87, 1.36)	0.003
Current asthma									
Yes	5.1	56.8 (48.2, 67.9)	0.725	0.67 (0.33, 1.01)	0.099	23.8 (17.4, 31.5)	0.065	1.03 (0.87, 1.27)	0.948
No	94.9	56.6 (47.0, 67.4)		0.74 (0.36, 1.04)		24.4 (18.2, 31.2)		1.04 (0.85, 1.32)	
Wheeze in past 12 months									
Yes	17.4	55.9 (46.7, 66.5)	0.064	0.62 (0.24, 0.98)	< 0.001	24.5 (18.3, 31.7)	0.099	1.03 (0.85, 1.27)	0.765
No	82.6	56.8 (47.1, 67.6)		0.76 (0.39, 1.06)		24.4 (18.1, 31.1)		1.05 (0.85, 1.32)	
Chronic bronchitis or emphysema									
Yes	7.8	56.3 (46.7, 67.7)	< 0.001	0.61 (0.23, 0.98)	< 0.001	23.0 (17.6, 31.0)	0.217	1.07 (0.88, 1.34)	< 0.001
No	92.2	56.6 (47.1, 67.4)		0.75 (0.37, 1.04)		24.5 (18.2, 31.2)		1.04 (0.85, 1.31)	

*Q1* 1st quartile, *Q3* 3rd quartile, *BMI* body mass index

*P*-values estimated using the Kruskal–Wallis *H* test for 2-level comparisons and the Dwass, Steel, Critchlow-Flinger analysis for 3 or more level comparisons

**Table 2:** Concentrations of vitamins A, C, D, and E (α- and γ-tocopherol) by characteristics of study participants, continuous NHANES (1999–2006)

Characteristics	% participants	Vitamin A (µg/dl)		Vitamin C (mg/dl)		25-OHD (ng/mL)		Vitamin E isoforms			
								α-Tocopherol (mg/dl)		γ-Tocopherol (µg/dl)	
		Median (Q1,Q3)	*P*	Median (Q1,Q3)	*P*	Median (Q1,Q3)	*P*	Median (Q1,Q3)	*P*	Median (Q1,Q3)	*P*
All participants	100	58.7 (48.5, 70.2)		0.95 (0.58, 1.24)		24.1 (18.5, 30.1)		1.17 (0.92, 1.52)		210 (137, 296)	
Age groups											
20–39 years	38.9	54.7 (45.0, 65.1)	Ref.	0.91 (0.57, 1.18)	Ref.	24.3 (18.3, 30.4)	Ref.	0.99 (0.82, 1.21)	Ref.	210 (151, 283)	Ref.
40–59 years	39.3	59.7 (49.7, 71.5)	< 0.001	0.92 (0.53, 1.18)	0.001	24.0 (18.7, 29.9)	< 0.001	1.24 (1.01, 1.58)	< 0.001	221 (142, 316)	< 0.001
60 + years	21.8	64.5 (54.5, 76.3)	< 0.001	1.10 (0.73, 1.43)	< 0.001	24.0 (18.6, 29.2)	0.015	1.47 (1.13, 1.98)	< 0.001	182 (104, 287)	< 0.001
Sex											
Men	48.2	62.6 (53.1, 73.4)	< 0.001	0.88 (0.50, 1.15)	< 0.001	24.4 (19.3, 29.8)	0.015	1.15 (0.91, 1.48)	< 0.001	213 (143, 298)	0.470
Women	51.8	54.8 (44.9, 66.3)		1.03 (0.67, 1.33)		23.7 (17.8, 30.2)		1.18 (0.94, 1.56)		206 (132, 293)	
Race/ethnicity											
Non-Hispanic Whites	71.9	61.0 (51.0, 72.5)	Ref.	0.98 (0.56, 1.27)	Ref.	26.0 (20.8, 31.4)	Ref.	1.22 (0.96, 1.60)	Ref.	206 (131, 294)	Ref.
Non-Hispanic Blacks	10.7	51.0 (41.1, 62.4)	< 0.001	0.88 (0.60, 1.14)	< 0.001	15.0 (11.2, 20.1)	< 0.001	0.98 (0.82, 1.21)	< 0.001	238 (170, 318)	< 0.001
Mexican Americans	7.6	52.1 (43.1, 62.0)	< 0.001	0.92 (0.65, 1.14)	< 0.001	20.7 (15.9, 25.6)	< 0.001	1.08 (0.88, 1.35)	< 0.001	214 (151, 296)	< 0.001
Other	9.9	55.0 (45.0, 65.2)	< 0.001	0.96 (0.64, 1.20)	0.132	20.8 (16.2, 26.3)	< 0.001	1.14 (0.90, 1.42)	< 0.001	201 (138, 278)	0.992
Income											
< $20,000	22.5	55.6 (45.1, 67.3)	< 0.001	0.88 (0.45, 1.17)	< 0.001	22.0 (15.9, 27.9)	< 0.001	1.07 (0.86, 1.41)	< 0.001	227 (152, 314)	< 0.001
$20,000 +	77.5	59.7 (49.5, 71.0)		0.97 (0.61, 1.25)		24.6 (19.2, 30.3)		1.19 (0.95, 1.55)		205 (133, 291)	
Education levels											
Below high school graduate	19.4	56.0 (45.5, 67.2)	< 0.001	0.84 (0.47, 1.15)	< 0.001	21.9 (15.9, 27.7)	< 0.001	1.10 (0.88, 1.40)	< 0.001	227 (153, 316)	< 0.001
High school graduate/Some college	55.9	58.8 (48.8, 69.9)	< 0.001	0.92 (0.53, 1.21)	< 0.001	24.3 (18.5, 30.2)	< 0.001	1.15 (0.91, 1.52)	< 0.001	220 (148, 306)	< 0.001
College graduate or higher	24.7	61.1 (50.6, 72.9)	Ref.	1.07 (0.81, 1.34)	Ref.	25.3 (20.5, 30.5)	Ref.	1.25 (1.01, 1.60)	Ref.	176 (111, 251)	Ref.
Cigarette smoke exposure											
No smoke exposure	28.9	58.0 (48.4, 70.2)	Ref.	1.07 (0.79, 1.33)	Ref.	24.4 (19.5, 29.9)	Ref.	1.24 (0.98, 1.63)	Ref.	186 (120, 268)	Ref.
Secondhand smoke exposure	21.4	56.1 (45.5, 67.4)	< 0.001	0.93 (0.58, 1.22)	< 0.001	22.6 (16.9, 29.1)	< 0.001	1.07 (0.87, 1.38)	< 0.001	228 (156, 310)	< 0.001
Past smokers (Quit > 5 years ago)	19.2	63.2 (53.2, 75.1)	< 0.001	1.04 (0.73, 1.33)	0.011	25.4 (19.9, 30.3)	0.200	1.33 (1.06, 1.77)	< 0.001	200 (116, 291)	0.020
Past smokers (Quit within last 5 years)	5.9	58.9 (48.6, 70.8)	0.985	0.94 (0.64, 1.18)	< 0.001	23.9 (19.2, 30.2)	0.709	1.14 (0.90, 1.47)	< 0.001	213 (148, 301)	< 0.001
Current smokers	24.6	57.8 (48.1, 68.6)	0.174	0.68 (0.29, 1.05)	< 0.001	23.6 (17.1, 30.2)	< 0.001	1.05 (0.86, 1.31)	< 0.001	226 (160, 311)	< 0.001
BMI											
Underweight	1.8	52.2 (40.6, 62.4)	< 0.001	1.04 (0.50, 1.34)	0.826	25.0 (18.5, 31.2)	0.029	1.01 (0.80, 1.25)	< 0.001	159 (102, 233)	0.097
Normal	32.1	57.7 (48.0, 69.8)	Ref...	1.06 (0.70, 1.34)	Ref.	26.3 (20.6, 32.2)	Ref.	1.11 (0.89, 1.44)	Ref.	171 (115, 237)	Ref.
Overweight	34.2	61.4 (51.2, 72.7)	< 0.001	0.99 (0.63, 1.25)	< 0.001	24.5 (19.4, 30.2)	< 0.001	1.23 (0.98, 1.62)	< 0.001	209 (139, 292)	< 0.001
Obese	31.9	57.0 (47.1, 68.4)	< 0.001	0.83 (0.47, 1.11)	< 0.001	21.6 (16.1, 27.3)	< 0.001	1.16 (0.93, 1.50)	0.004	256 (179, 354)	< 0.001
Alcohol consumption											
Heavy drinker	9.9	64.9 (55.0, 77.8)	< 0.001	0.93 (0.48, 1.23)	< 0.001	25.5 (19.9, 31.3)	< 0.001	1.17 (0.93, 1.52)	0.004	207 (135, 291)	0.025
Light/moderate drinker	62.7	59.2 (49.2, 70.2)	< 0.001	0.95 (0.59, 1.23)	0.001	24.7 (19.4, 30.4)	< 0.001	1.16 (0.93, 1.51)	0.007	207 (136, 290)	< 0.001
Non-drinker	27.4	55.7 (45.4, 67.5)	Ref.	0.98 (0.59, 1.29)	Ref.	22.4 (16.7, 28.1)	Ref.	1.18 (0.92, 1.57)	Ref.	216 (140, 310)	Ref.
Taking vitamin supplements											
Yes	53.5	61.5 (51.0, 73.4)	< 0.001	1.09 (0.82, 1.37)	< 0.001	25.5 (20.6, 31.2)	< 0.001	1.35 (1.06, 1.80)	< 0.001	170 (107, 254)	< 0.001
No	46.5	55.5 (46.1, 66.3)		0.74 (0.36, 1.05)		22.2 (15.9, 28.3)		1.01 (0.84, 1.22)		247 (184, 335)	
Caloric intake tertiles											
3rd (higher)	33.2	59.9 (50.5, 71.1)	0.002	0.90 (0.54, 1.18)	< 0.001	24.9 (19.6, 30.3)	< 0.001	1.11 (0.89, 1.44)	< 0.001	222 (151, 302)	< 0.001
2nd	34.1	58.9 (48.5, 70.4)	0.111	1.00 (0.61, 1.26)	0.980	24.0 (18.5, 30.2)	< 0.001	1.20 (0.94, 1.57)	0.932	205 (134, 292)	0.824
1st (lower)	32.7	57.3 (46.9, 69.2)	Ref.	0.98 (0.61, 1.30)	Ref.	23.5 (17.7, 29.3)	Ref.	1.19 (0.94, 1.58)	Ref	202 (127, 293)	Ref
General health status											
Excellent or very good	52.0	59.4 (49.3, 70.6)	Ref.	1.03 (0.70, 1.30)	Ref.	25.6 (20.4, 31.2)	Ref.	1.18 (0.93, 1.53)	Ref.	199 (128, 276)	Ref.
Good	31.4	58.1 (48.0, 69.4)	0.228	0.90 (0.52, 1.19)	< 0.001	23.0 (17.6, 29.2)	< 0.001	1.15 (0.92, 1.51)	0.008	218 (146, 305)	< 0.001
Fair or poor	16.6	57.9 (46.5, 70.7)	0.167	0.82 (0.44, 1.14)	< 0.001	20.9 (15.5, 26.7)	< 0.001	1.15 (0.91, 1.50)	0.050	237 (154, 337)	< 0.001
Current asthma											
Yes	6.1	59.3 (48.7, 70.7)	0.540	0.90 (0.53, 1.22)	0.028	23.3 (17.9, 29.5)	0.321	1.15 (0.91, 1.47)	0.043	213 (146, 296)	0.121
No	93.9	58.7 (48.6, 70.2)		0.96 (0.59, 1.24)		24.2 (18.6, 30.1)		1.17 (0.93, 1.53)		209 (137, 296)	
Wheeze in past 12 months											
Yes	15.5	59.0 (48.7, 70.5)	0.030	0.85 (0.42, 1.15)	< 0.001	23.4 (17.5, 29.4)	0.078	1.14 (0.91, 1.48)	0.001	230 (158, 324)	< 0.001
No	84.5	58.7 (48.5, 70.2)		0.98 (0.62, 1.25)		24.3 (18.7, 30.2)		1.17 (0.93, 1.53)		206 (134, 291)	
Chronic bronchitis or emphysema											
Yes	6.9	61.1 (48.5, 73.1)	< 0.001	0.76 (0.36, 1.17)	< 0.001	23.3 (17.9, 29.2)	0.339	1.21 (0.94, 1.59)	0.057	234 (152, 332)	0.002
No	93.1	58.5 (48.5, 70.1)		0.97 (0.60, 1.24)		24.2 (18.5, 30.1)		1.16 (0.92, 1.52)		208 (137, 294)	

*Q1* 1st quartile, *Q3* 3rd quartile, *BMI* body mass index

*P*-values estimated using the Kruskal–Wallis H test for 2-level comparisons and the Dwass, Steel, Critchlow-Flinger analysis for 3 or more level comparisons

### Serum antioxidants—variation by participant characteristics

Participant serum antioxidant vitamin levels varied notably by sociodemographic and behavioral characteristics (Tables 1 and 2). In both surveys, vitamin (A, C, 25-OHD, and α-tocopherol) levels were found to be highest in non-Hispanic Whites and among more affluent and educated adults, whereas lowest levels were found in non-Hispanic Blacks and those with lower socioeconomic status. Levels of vitamins A, C, and α-tocopherol tended to increase with age, whereas age-specific patterns were different and less consistent for 25-OHD and γ-tocopherol. Females had higher levels of vitamin C and α-tocopherol, while levels of vitamin A and 25-OHD were higher in males. Vitamin levels varied by BMI, showing similar variation across the survey cycles, but slightly different patterns for each vitamin. Vitamin levels also differed by smoking exposure and alcohol consumption. Among current smokers and those who reported exposure to secondhand smoke, vitamin levels tended to be lower than those who did not report cigarette smoke exposure. While non-drinkers had the highest vitamin C and α-tocopherol levels, they had lowest levels of vitamin A and 25-OHD. Vitamin levels (A, C, 25-OHD, and α-tocopherol) were consistently higher among participants who reported taking vitamin supplements in the past month than those who did not report supplemental vitamin use. Vitamin A and 25-OHD levels increased with increasing caloric intake, whereas those with the highest caloric intake had the lowest levels of vitamin C and α-tocopherol. Vitamin C and 25-OHD levels decreased with decreasing health status. The levels of the vitamin E isoforms showed opposing patterns in relation to most participant characteristics; however, both α-tocopherol and γ-tocopherol tended to be higher among non-drinkers and overweight and obese individuals, and variation by age and sex was less consistent.

### Serum vitamin A and respiratory morbidity and mortality

**Fig. 1:**
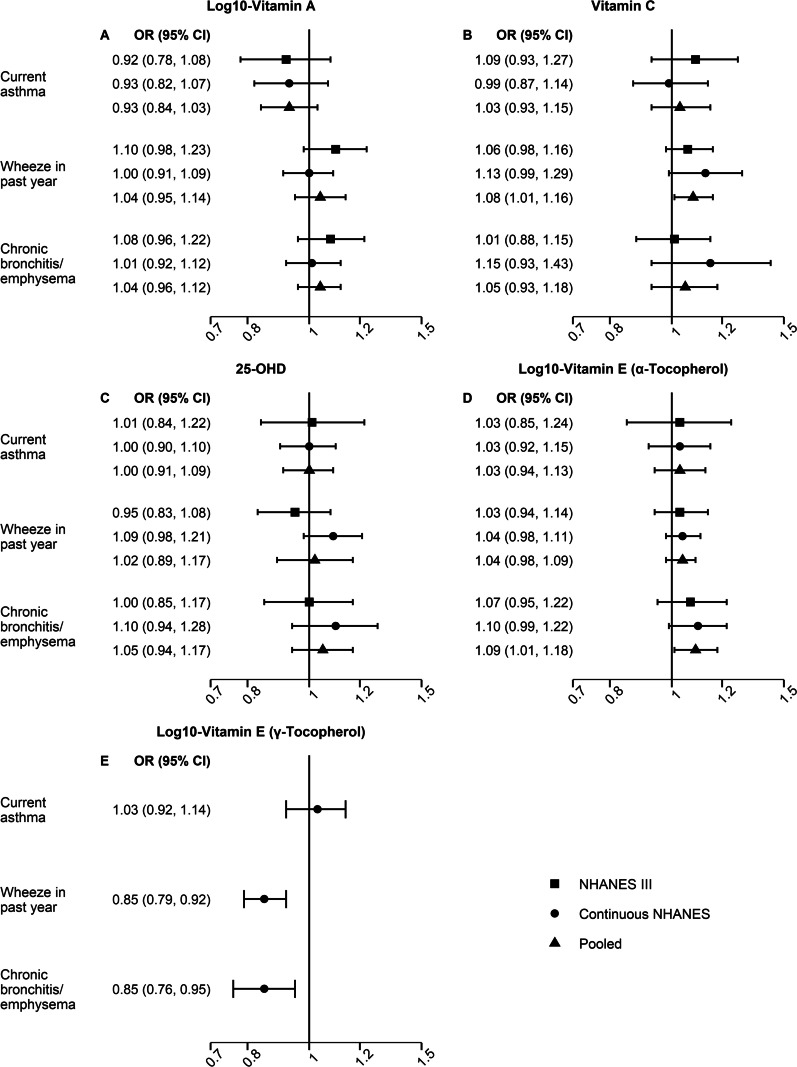
Serum vitamin levels and respiratory morbidity. Forest plots for the association of an interquartile range decrease in serum levels of log_10_-vitamin A (**A**), vitamin C (**B**), 25-OHD (**C**), and log_10_-vitamin E [α-tocopherol (**D**), γ-tocopherol (**E**)] with respiratory morbidity [NHANES III (1988–1994), continuous NHANES (1999–2006), and pooled estimates]. Data on γ- tocopherol isoform of vitamin E were available only for continuous NHANES. The models were adjusted for age, gender, race/ethnicity, income, educational attainment, use of any vitamin supplements in the previous month, cigarette smoking and exposure to cigarette smoke, pack-years of cigarette smoking, BMI, alcohol consumption, caloric intake, general health status, and survey cycle (only continuous NHANES). Models for serum 25-OHD were further adjusted for season of blood collection, and vitamin E models were additionally adjusted for total serum cholesterol

**Fig. 2:**
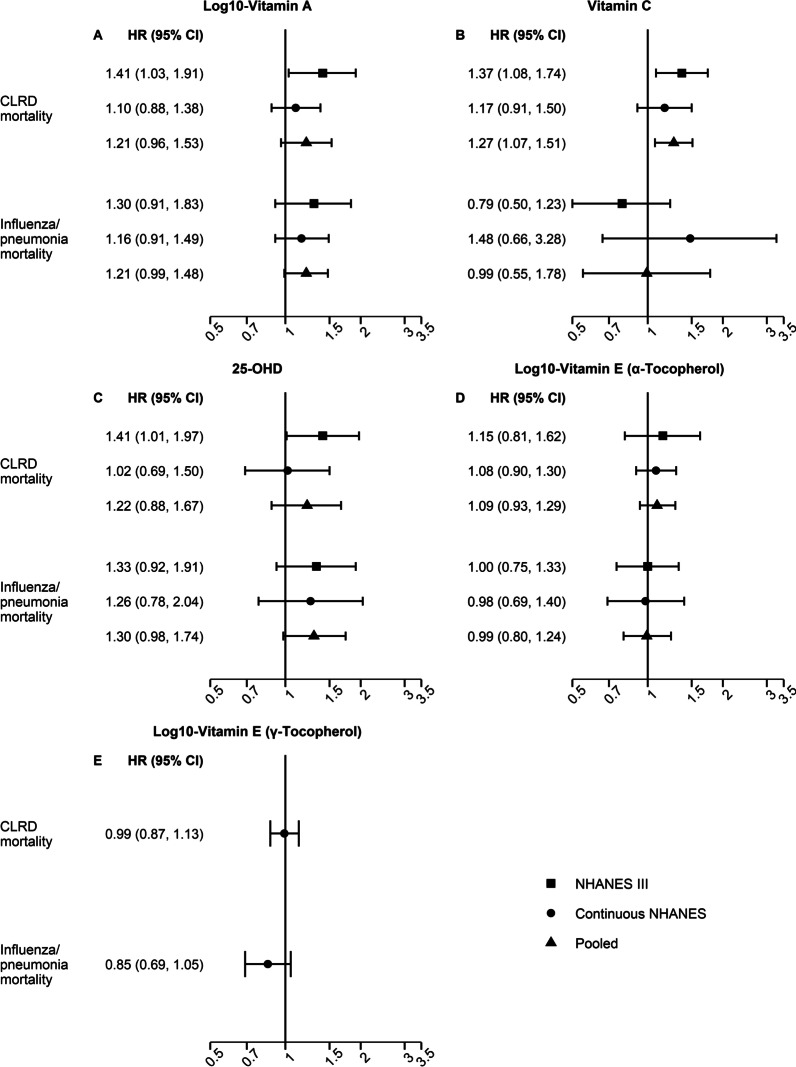
Serum vitamin levels and respiratory mortality. Forest plots for the association of an interquartile range decrease in serum levels of log_10_-vitamin A (**A**), vitamin C (**B**), 25-OHD (**C**), and log_10_-vitamin E [α-tocopherol (**D**), γ-tocopherol (**E**)] with respiratory mortality [NHANES III (1988–1994), continuous NHANES (1999–2006), and pooled estimates]. Data on γ- tocopherol isoform of vitamin E were available only for continuous NHANES. The models were adjusted for age, gender, race/ethnicity, income, educational attainment, use of any vitamin supplements in the previous month, cigarette smoking and exposure to cigarette smoke, pack-years of cigarette smoking, BMI, alcohol consumption, caloric intake, general health status, and survey cycle (only continuous NHANES). Models for serum 25-OHD were further adjusted for season of blood collection, and vitamin E models were additionally adjusted for total serum cholesterol

Respiratory morbidity was not associated with serum vitamin A levels (Fig. 1A). Lower serum vitamin A concentrations were associated with increased mortality from influenza/pneumonia (pooled aHR: 1.21, 95% CI: 0.99–1.48), though the association did not reach statistical significance (Fig. 2A). There was no strong evidence suggesting that the pooled estimates were modified by vitamin supplement use or smoking (Additional file 1: Table S1). However, the cohort-specific results showed significant heterogeneity for chronic bronchitis/emphysema- and CLRD-related outcomes by vitamin supplement use and smoking (Additional file 1: Table S4). For example, lower serum vitamin A was associated with increased CLRD mortality among those who reported no vitamin supplement use (aHR: 2.03, 95% CI: 1.58–2.61) and among smokers (aHR: 1.61, 95% CI: 1.21–2.15) in the NHANES III cohort, but not in the continuous NHANES cohort (*P*_heterogeneity_: 0.010 and 0.039, respectively; Additional file 1: Table S4).

### Serum vitamin C and respiratory morbidity and mortality

Lower serum vitamin C concentrations were associated with higher odds of wheeze in the past 12 months (pooled aOR: 1.08, 95% CI: 1.01–1.16) and increased mortality from CLRD (pooled aHR: 1.27, 95% CI: 1.07–1.51) (Figs. 1B and 2B). The associations between serum vitamin C levels and respiratory morbidity and mortality were not modified by supplemental vitamin use or smoking (Additional file 1: Tables S1–S3). However, cohort-specific results showed variation in stratified analyses of vitamin C levels and influenza/pneumonia mortality among those taking supplements (*P*_heterogeneity_ = 0.037) (Additional file 1: Table S4). While vitamin C deficiency became less common over time, it was associated with increased mortality from influenza/pneumonia in pooled analysis (pooled aHR: 2.12, 95% CI: 1.12–4.02; Additional file 1: Table S5). Vitamin C deficiency also increased the odds of chronic bronchitis/emphysema (aOR: 1.81, 95% CI: 1.35–2.43; Additional file 1: Table S5), albeit only among continuous NHANES participants due to significant heterogeneity between the two study populations.

### Serum 25-OHD and respiratory morbidity and mortality

**Fig. 3:**
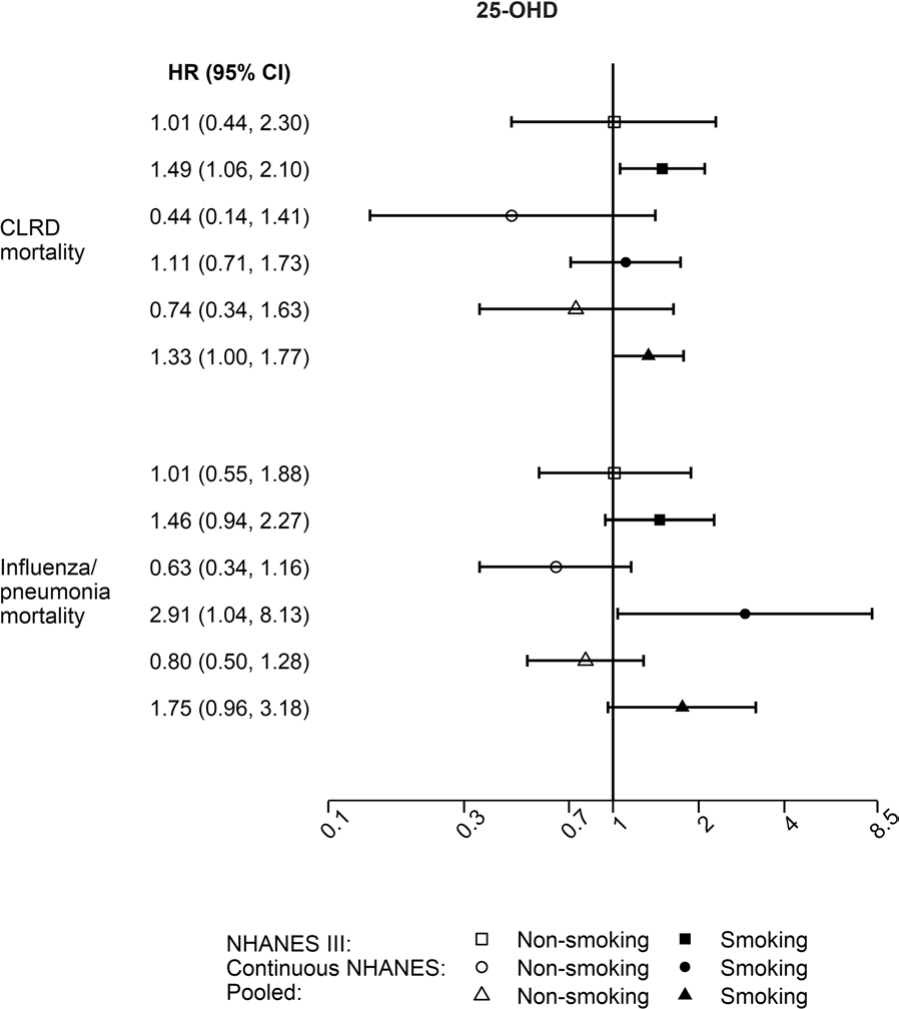
Serum 25-OHD and respiratory mortality by smoking. Forest plots for the associations between lower serum 25-OHD and respiratory mortality by smoking [NHANES III (1988–1994), continuous NHANES (1999–2006), and pooled estimates]. The models were adjusted for age, gender, race/ethnicity, income, educational attainment, use of any vitamin supplements in the previous month, cigarette smoking and exposure to cigarette smoke, pack-years of cigarette smoking, BMI, alcohol consumption, caloric intake, general health status, season of blood collection, and survey cycle (only continuous NHANES). *P*_interaction_ for 25-OHD*smoking < 0.01

Lower serum 25-OHD concentrations were not associated with respiratory morbidity (Fig. 1C), and none of these associations were influenced by supplemental vitamin use or smoking (Additional file 1: Tables S1–S3). The association between serum 25-OHD levels and mortality from influenza/pneumonia was modified by smoking (*P*_interaction_ < 0.01; Additional file 1: Table S1). Increased mortality was not observed in non-smokers (pooled aHR: 0.80, 95% CI: 0.50–1.28), but influenza/pneumonia mortality increased among smokers (pooled aHR: 1.75, 95% CI: 0.96–3.18), though the association remained marginally significant (Fig. 3, Additional file 1: Table S3). A similar pattern was observed for CLRD mortality (Fig. 3); however, the interaction did not reach the threshold for statistical significance (Additional file 1: Table S1). Lower serum 25-OHD was associated with increased CLRD mortality (pooled aHR: 1.33, 95% CI: 1.00–1.77) among smokers, but not in non-smokers (pooled aHR: 0.74, 95% CI: 0.34–1.63) (Fig. 3, Additional file 1: Table S3). Serum 25-OHD insufficiency (< 0.20 ng/mL) was associated with increased mortality from influenza/pneumonia (pooled aHR: 1.67, 95% CI: 1.11–2.53) (Additional file 1: Table S5). The association between 25-OHD deficiency (< 12 ng/mL) and respiratory outcomes could not be estimated due to sparse data. No significant heterogeneity between NHANES III and continuous NHANES was observed for any of the 25-OHD analyses.

### Serum vitamin E and respiratory morbidity and mortality

**Fig. 4:**
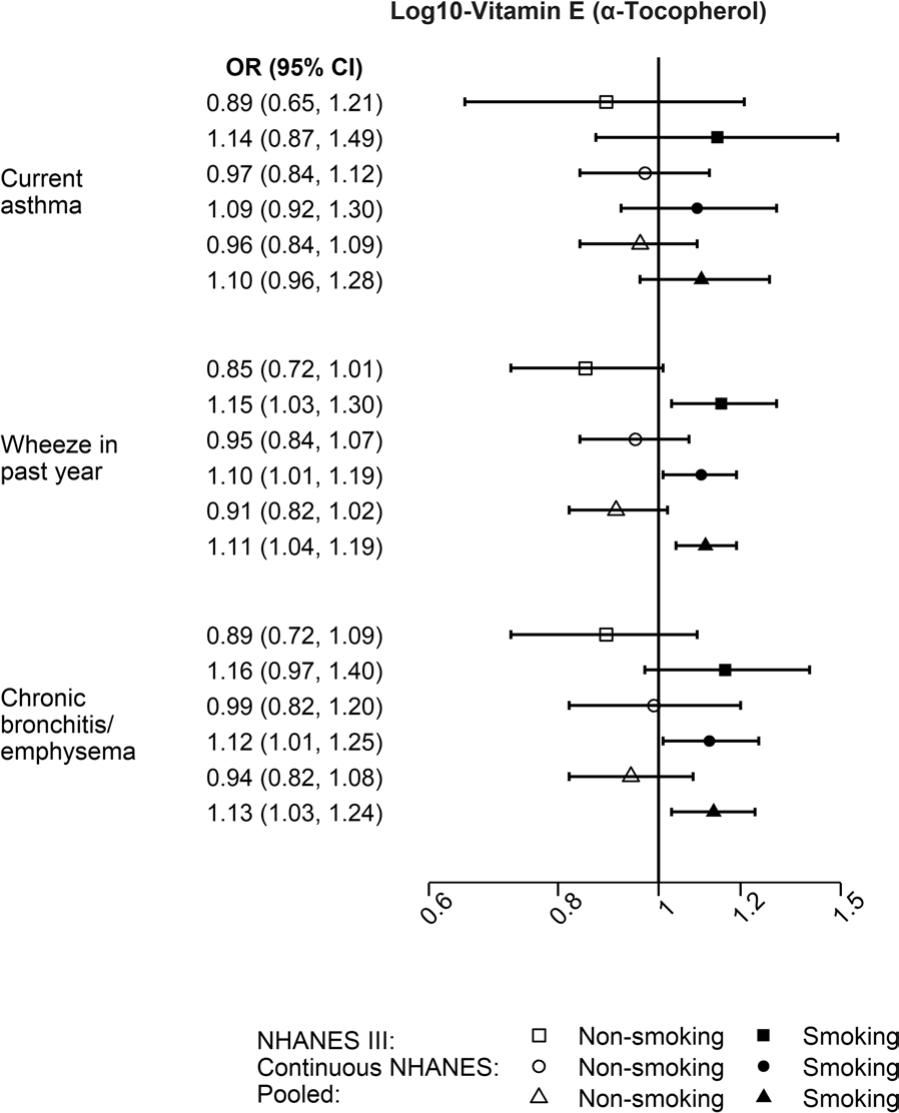
Serum α-tocopherol and respiratory morbidity by smoking. Forest plots for the associations between lower serum log_10_- α-tocopherol vitamin E and respiratory mortality by smoking [NHANES III (1988–1994), continuous NHANES (1999–2006), and pooled estimates]. The models were adjusted for age, gender, race/ethnicity, income, educational attainment, use of any vitamin supplements in the previous month, cigarette smoking and exposure to cigarette smoke, pack-years of cigarette smoking, BMI, alcohol consumption, caloric intake, general health status, total serum cholesterol, and survey cycle (only continuous NHANES). *P*_interaction_ for vitamin E*smoking < 0.01

The associations of serum α-tocopherol vitamin E with wheeze in the past 12 month and chronic bronchitis/emphysema varied by smoking (*P*_interaction_ < 0.01). Lower serum α-tocopherol vitamin E was associated with increased odds of wheeze in the past 12 months (pooled aOR: 1.11, 95% CI: 1.04–1.19) and chronic bronchitis/emphysema (pooled aOR: 1.13, 95% CI: 1.03–1.24) in smokers, but not in non-smokers (pooled aOR: 0.91, 95% CI: 0.82–1.02 for wheeze in the past 12 months; pooled aOR: 0.94, 95% CI: 0.82–1.08 for chronic bronchitis/emphysema) (Fig. 4, Additional file 1: Tables S1 and S3). The association between serum α-tocopherol and respiratory outcomes was not modified by vitamin supplement use (Additional file 1: Tables S1–S2). No significant heterogeneity between NHANES III and continuous NHANES was observed for any of the analyses. In contrast to α-tocopherol, lower serum levels of γ-tocopherol vitamin E were associated with lower odds of wheeze in the past 12 months (aOR: 0.85, 95% CI: 0.79–0.92) and chronic bronchitis/ emphysema (aOR: 0.85, 95% CI: 0.76–0.95) (Fig. 1E). No association was found between either of the serum vitamin E isoforms and respiratory mortality (Fig. 2E).

## Discussion

Our large, nationally representative analysis showed that lower serum levels of vitamins C and α-tocopherol were associated with an increase in wheeze and chronic bronchitis/emphysema, although the effects of α-tocopherol were primarily observed in smokers. Consistent with recent reports [6, 7], serum α- and γ-tocopherol had contrasting effects on respiratory outcomes. Lower levels of serum vitamin A, C, and 25-OHD were associated with increased mortality from CLRD and influenza/pneumonia. Smoking, which is known to deplete serum vitamin antioxidant levels [9], not only modified the association between serum α-tocopherol and respiratory morbidity, but also the association between serum 25-OHD and respiratory mortality. Clinical thresholds established for vitamin C deficiency and 25-OHD insufficiency were most consistently associated with increased respiratory mortality from influenza/pneumonia. Although serum antioxidant vitamin levels were significantly higher among those who used vitamin supplements compared to those who did not, there was no clear statistical evidence of effect modification by supplement use. None of the asthma-related associations reached statistical significance in our study, in contrast to recently reported beneficial effects of antioxidant vitamins on asthma [16].

Vitamin A plays an important role in the proliferation and maintenance of epithelial cells in the lung [39, 40]. Evidence from animal models has long suggested a possible link between vitamin A and COPD [41]. For instance, weanling rats fed with a diet poor in vitamin A for 6 weeks have been shown to develop emphysema independently of exposure to cigarette smoke [42]. Other animal studies have reproduced these findings and confirmed that retinoic acid could reduce the risk of COPD [41]. Yet, epidemiological studies investigating the relationship between vitamin A and respiratory disorders in adults have reported conflicting results [3, 9, 15]. In our study, vitamin A levels were not associated with respiratory morbidity, but our longitudinal analysis demonstrated that a 30-percent decrease in vitamin A levels was associated with a 103% increase in CLRD mortality, albeit only among NHANES III participants who did not use vitamin supplements. Decreasing vitamin A levels were also associated with a 61% increase in CLRD mortality among smoking NHANES III participants. Although vitamin A deficiency was uncommon among U.S. adults, vitamin A levels were significantly lower among participants who did not use vitamin supplements than in those who reported supplemental vitamin use. Insufficient vitamin A intake and vitamin A deficiency have been associated with histopathological changes within the respiratory tract that can disrupt physiological functions and lead to severe tissue dysfunction in the lung [39, 40]. In addition to its critical role in the maintenance of healthy immune responses and epithelial integrity, vitamin A can affect pathogen control [39, 40, 43]. In NHANES, lower vitamin A levels were associated with increased mortality risk from influenza/pneumonia. Although this association did not quite reach statistical significance, our results support previous findings that have shown vitamin A to influence host response to influenza virus infection [43].

Vitamin C, the most extensively studied antioxidant, is known to have anti-inflammatory and immunomodulatory properties. It is a potent antioxidant in airway surface liquid and protects against endogenous and exogenous oxidants, by supporting epithelial barrier function and affecting functions of both innate and adaptive immune cells [44, 45]. We showed that a 0.65 mg/dl decrease in serum vitamin C concentration increased the odds of wheeze by 8% among the study participants. Our finding of an inverse association between serum vitamin C and wheeze is consistent with results from an older NHANES II study (1976–1980) and studies that have examined dietary vitamin C intake in relation to wheeze and/or lung function, although conflicting data have also been reported [9, 17, 46, 47]. Vitamin C deficiency was not associated with chronic bronchitis/emphysema in NHANES III but increased the odds of having chronic bronchitis/emphysema by 81% in the continuous NHANES, agreeing with previous studies [47, 48]. Interestingly, a recent mouse model has suggested that vitamin C may not only prevent smoke-induced emphysema but could also restore emphysematous lungs [49], highlighting the importance of vitamin C in COPD. Although the intake and serum concentrations of vitamin C have been associated with all-cause mortality in the general population and among adults with obstructive lung function [20, 50], limited national-level data exist on how vitamin C relates to respiratory mortality [21]. We found that a 0.65 mg/dl decrease in serum vitamin C levels was associated with 27% higher CLRD mortality. The association between vitamin C deficiency and mortality from influenza/pneumonia was even more pronounced, with a 112% increase, consistent with previous studies that have linked vitamin C deficiency to pneumonia and other respiratory infections [44, 45].

Vitamin D has been featured prominently in respiratory research in the past decades due to its immunoregulatory, anti-inflammatory, and antioxidant properties [51]. Vitamin D is vital for many physiological functions, as it regulates a diverse set of genes associated with cell proliferation and differentiation, cell control, apoptosis, and host defense mechanisms [10, 52]. Vitamin D is thought to play an essential role in tissue remodeling and in innate and adaptive immune responses in the lung. Despite increasing evidence of inverse associations of serum vitamin D levels with asthma and COPD morbidity [4, 10, 53, 54], limited and conflicting data exist on whether vitamin D levels relate to respiratory mortality [55–58]. In our study, lower levels of 25-OHD were associated with respiratory mortality, but not morbidity. Vitamin D insufficiency and smoking, which often coexist, were consistently associated with mortality. Among smokers, a 12 ng/mL decrease in 25-OHD was associated with a 33% and a 75% higher mortality risk from CLRD and influenza/pneumonia, respectively. The magnitude of the association between 25-OHD insufficiency and influenza/pneumonia was similar. This supports a growing number of reports that have linked lower vitamin D levels with the risk and severity of respiratory tract infections, including influenza, and more recently COVID-19 [10, 52].

Cigarette smoking is a major cause of oxidative stress, as it generates free radicals (some of which are from compounds in the smoke) and induces significant inflammatory responses involving neutrophils and macrophages [59]. Increased oxidative stress due to smoking causes tissue damage through lipid peroxidation which has been shown to be reduced by vitamin E, a free radical scavenger found in lipid membranes and extracellular lung fluids [18, 59]. Although cigarette smoke-related oxidants can deplete plasma vitamin E in vitro, not all studies have found notable differences in smokers’ and non-smokers’ plasma vitamin E levels [59]. In our study, the lowest levels of α-tocopherol were consistently found in current smokers. Our modeling results showed that a 30-percent decrease in α-tocopherol levels increased the odds of wheeze by 11% and chronic bronchitis/emphysema by 13% in smokers but not in non-smokers. In contrast, lower serum γ-tocopherol levels were associated with lower odds of wheeze and chronic bronchitis/emphysema, reducing the odds by 15%. There is increasing evidence that α- and γ-tocopherol isoforms have opposing effects on lung inflammation and disease [6, 7]. Our results support the paradigm that α-tocopherol may be anti-inflammatory and γ-tocopherol pro-inflammatory in nature [6, 16].

We acknowledge that our study has limitations. NHANES data collection is cross-sectional in nature, which precludes establishing temporal relationships between serum antioxidant vitamins and respiratory morbidity. All morbidity outcomes were defined by self-report without objective assessment of pulmonary function, though self-reported asthma has shown good accuracy when compared to medical records [60]. Because the prevalence of most vitamin deficiencies in this population was low, we were only able to evaluate vitamin C deficiency in relation to respiratory morbidity and mortality. Caution, however, is warranted when interpreting the mortality related findings as the results for the continuous NHANES are based on relatively small numbers of deaths. Due to the survey design, serum antioxidant vitamins and covariates were only measured at enrollment, and thus we could not include information on time-varying covariates or diagnostic follow-up data. Potential sources of heterogeneity in some of the cohort-specific estimates remain unclear, but differences in study population characteristics and other time-varying factors, such as changes in nutritional enrichment and fortification of foods [61], might have contributed to the observed effects. All our models, however, were adjusted for a comprehensive set of potential confounders, and our longitudinal analysis of up to 27 years accounted for competing risks of mortality.

Despite these limitations, our study has notable strengths. Ours is the largest and most comprehensive epidemiological study on serum antioxidant vitamins and respiratory morbidity and mortality in adults conducted to date. Only few large-scale studies have investigated serum antioxidant vitamin levels in relation to respiratory mortality at the national level [20, 21]. The use of two large, nationally representative samples of adults increases the generalizability of our results and likely increased the precision of our reported effect estimates compared to prior studies. Additionally, because vital status was assessed prospectively for all the participants via linkage to national mortality data, concerns of selection bias due to loss to follow-up are minimal. NHANES data collection and survey procedures also followed standardized protocols and underwent rigorous quality assurance procedures.

Future prospective studies with repeated measures of serum antioxidant vitamins and covariates are needed to confirm our results. Additional research is also warranted to better understand underlying mechanisms of the findings, especially in susceptible groups, including smokers and those with vitamin deficiencies or insufficiencies. Given that respiratory diseases are major causes of morbidity and mortality worldwide [16, 17, 21], it is crucial to further elucidate the role of antioxidant vitamins in respiratory health in longitudinal studies and randomized controlled clinical trials, some of which to date have reported mixed findings and linked vitamin supplement use with an unexpected increase in mortality [62].

## Conclusions

Our nationally representative results demonstrated that lower serum levels of vitamins A, C, D, and α-tocopherol vitamin E were associated with higher respiratory morbidity and mortality in the U.S. adult population, whereas opposite effects were observed for serum γ-tocopherol vitamin E. Our findings highlight the importance of antioxidant vitamins in respiratory health, suggesting that assessing serum levels of antioxidant vitamins could be helpful in identifying individuals who might be at higher risk for respiratory morbidity and mortality.

## Supplementary Information

**Additional file 1: Figure S1**. Flow chart for participants.** Table S1**. Interactions for the associations between serum vitamins and respiratory morbidity and mortality: *P*-values for effect modification by vitamin supplement use and smoking.** Table S2**. Associations between lower serum antioxidant vitamins and respiratory morbidity and mortality by use of vitamin supplements [NHANES III (1988–1994) and continuous NHANES (1999–2006)].** Table S3**. Associations between lower serum antioxidant vitamins and respiratory morbidity and mortality by smoking status [NHANES III (1988–1994) and continuous NHANES (1999-2006)].** Table S4. **Cohort specific estimates in models with significant heterogeneity.** Table S5**. Association of vitamin C deficiency and vitamin D (25-OHD) insufficiency with respiratory morbidity and mortality [NHANES III (1988–1994) and continuous NHANES (1999–2006)].

## Data Availability

All datasets are publicly available and can be accessed through NHANES websites (https://www.cdc.gov/nchs/nhanes/index.htm) [22].

## Abbreviations

aHR: Adjusted hazard ratio
aOR: Adjusted odds ratio
CDC: Centers for Disease Control and Prevention
CLRD: Chronic lower respiratory disease
COPD: Chronic obstructive pulmonary disease
COVID-19: Coronavirus disease 2019
ICD10: 10th revision of the International Classification of Diseases
NCHS: National Center for Health Statistics
NHANES: National Health and Nutrition Examination Survey
RNS: Reactive nitrogen species
ROS: Reactive oxygen species
25-OHD: 25-Hydroxyvitamin D
