# Supplementary material for: Serum antioxidant vitamins and respiratory morbidity and mortality: a pooled analysis
Source: Respir Res. 2022 Jun 9;23:150. doi: 10.1186/s12931-022-02059-w (PMC9178544; doi:10.1186/s12931-022-02059-w)
Supplement: Supplementary file 1 — Additional file 1: Figure S1. Flow chart for participants. Table S1. Interactions for the associations between serum vitamins and respiratory morbidity and mortality: P-values for effect modification by vitamin supplement use and smoking. Table S2. Associations between lower serum antioxidant vitamins and respiratory morbidity and mortality by use of vitamin supplements [NHANES III (1988–1994) and continuous NHANES (1999–2006)]. Table S3. Associations between lower serum antioxidant vitamins and respiratory morbidity and mortality by smoking status [NHANES III (1988–1994) and continuous NHANES (1999-2006)]. Table S4. Cohort specific estimates in models with significant heterogeneity. Table S5. Association of vitamin C deficiency and vitamin D (25-OHD) insufficiency with respiratory morbidity and mortality [NHANES III (1988–1994) and continuous NHANES (1999–2006)]. [file 12931_2022_2059_MOESM1_ESM.docx]

**Online Additional file 1**

Salo et al., Serum Antioxidant Vitamins and Respiratory Morbidity and Mortality: A Pooled Analysis

**
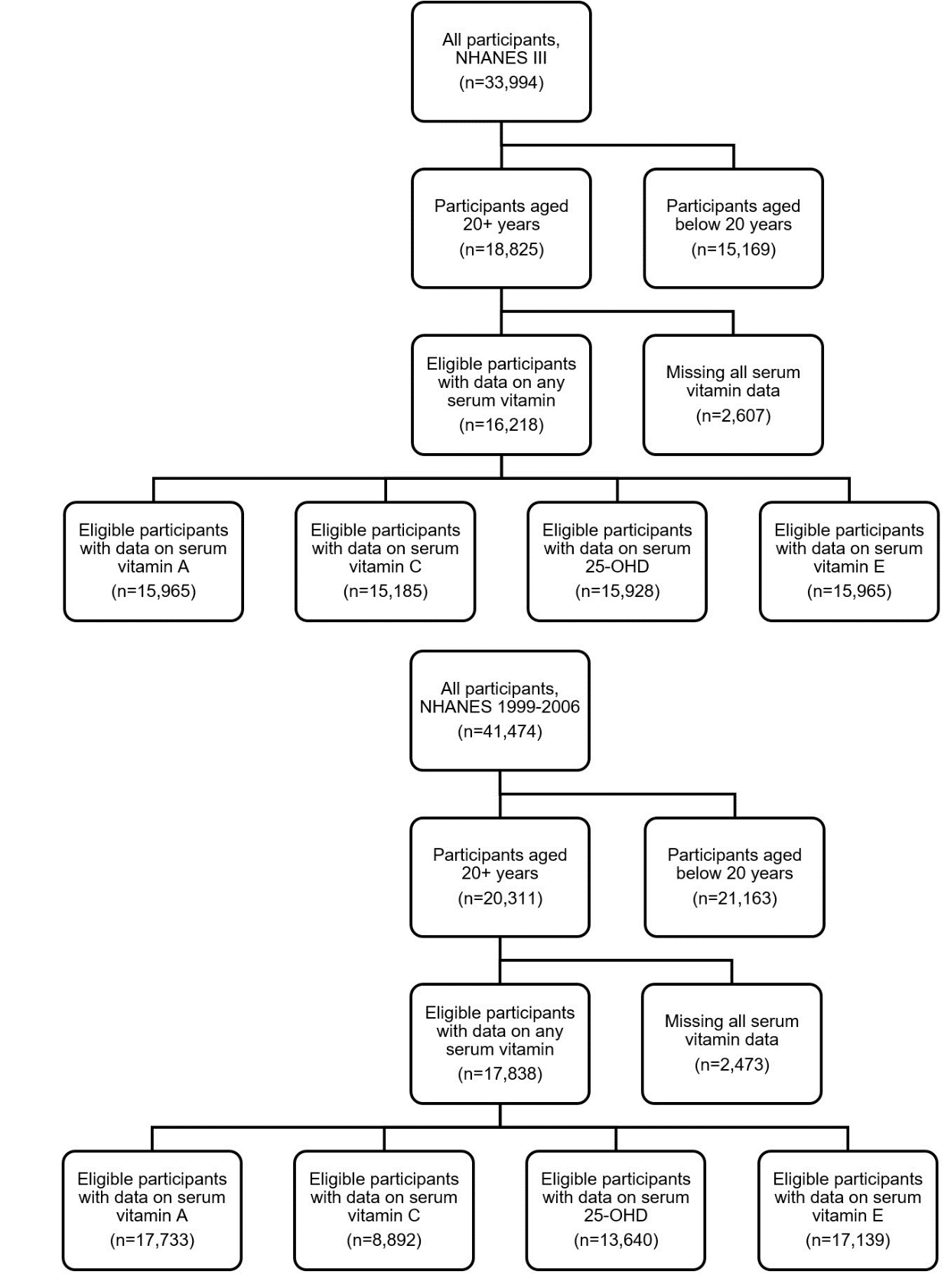
**

**Additional file 1: Figure S1**: Flow chart for participants

**Additional file 1: Table S1**: Interactions for the associations between serum vitamins and respiratory morbidity and mortality: *P*-values for effect modification by vitamin supplement use and smoking

|  | Respiratory Morbidity | | | |  | | Respiratory Mortality | | |
| --- | --- | --- | --- | --- | --- | --- | --- | --- | --- |
|  | Current asthma | Wheeze | Chronic bronchitis or emphysema |  | | Mortality from CLRD | | Mortality from influenza/  pneumonia |  |
| **Supplement Use*Vitamins** |  |  |  |  | |  | |  |  |
| Supplement Use*Vitamin A | 0.95 | 0.65 | 0.15 |  | | 0.31 | | 0.28 |  |
| Supplement Use *Vitamin C | 0.06 | 0.91 | 0.21 |  | | 0.72 | | 0.12 |  |
| Supplement Use *Vitamin D^a^ | 0.07 | 0.26 | 0.40 |  | | 0.89 | | 0.93 |  |
| Supplement Use *Vitamin E^b^ | 0.48 | 0.36 | 0.11 |  | | 0.22 | | 0.83 |  |
| **Smoking*Vitamins** |  |  |  |  | |  | |  |  |
| Smoking*Vitamin A | 0.77 | 0.07 | 0.11 |  | | 0.41 | | 0.34 |  |
| Smoking*Vitamin C | 0.74 | 0.35 | 0.10 |  | | 0.67 | | 0.25 |  |
| Smoking*Vitamin D^a^ | 0.54 | 0.85 | 0.76 |  | | 0.13 | | <0.01 |  |
| Smoking*Vitamin E^b^ | 0.85 | <0.01 | <0.01 |  | | 0.47 | | 0.46 |  |

^a^ Vitamin D: 25-OHD

^b^ Vitamin E: α-tocopherol

Models adjusted for age, sex, race/ethnicity, income, education level, use of vitamin supplements, cigarette smoking and exposure to cigarette smoke, pack-years of cigarette smoking, BMI, alcohol consumption, caloric intake, general health status, survey cycle (continuous NHANES only), and the interaction term of interest. Vitamin D models were additionally adjusted for season of collection, and vitamin E models were additionally adjusted for total serum cholesterol. Effect estimates are shown in Supplementary Table 2 (supplemental vitamin use) and Supplementary Table 3 (smoking status).

**Additional file 1: Table S2**: Associations between lower serum antioxidant vitamins and respiratory morbidity and mortality by use of vitamin supplements [NHANES III (1988-1994) and continuous NHANES (1999-2006)]

|  | Interquartile Decrease in log_10_-Vitamin A | | |  | Interquartile Decrease in Vitamin C | | |
| --- | --- | --- | --- | --- | --- | --- | --- |
|  | n/N | Effect size (95% CI) | *P*-value |  | n/N | Effect size (95% CI) | *P*-value |
| **In participants not taking supplements** |  |  |  |  |  |  |  |
| ***Respiratory morbidity ^a^*** |  |  |  |  |  |  |  |
| Current asthma | 737/15,269 | 0.91 (0.79-1.04) | 0.181 |  | 618/11,723 | 1.12 (0.96-1.31) | 0.159 |
| Wheeze in past 12 months | 2,130/15,471 | 1.06 (0.97-1.16) | 0.175 |  | 1,658/11,750 | 1.12 (0.96-1.31) | 0.158 |
| Chronic bronchitis or emphysema | 967/15,437 | 1.11 (1.00-1.25) | 0.055 |  | 770/11,723 | 1.11 (0.90-1.37) | 0.322 |
| ***Respiratory mortality ^b^*** |  |  |  |  |  |  |  |
| Mortality from CLRD | 185/15,429 | 1.57 (0.93-2.65)***^c^*** | 0.088 |  | 147/11,718 | 1.35 (0.74-2.47) | 0.328 |
| Mortality from Influenza/pneumonia | 100/15,429 | 1.10 (0.76-1.60) | 0.617 |  | 85/11,718 | 1.43 (0.80-2.55) | 0.234 |
| **In participants taking supplements** |  |  |  |  |  |  |  |
| ***Respiratory morbidity ^a^*** |  |  |  |  |  |  |  |
| Current asthma | 716/12,546 | 0.94 (0.81-1.08) | 0.391 |  | 575/8,930 | 0.95 (0.77-1.18) | 0.660 |
| Wheeze in past 12 months | 1,934/12,762 | 1.00 (0.90-1.11) | 0.983 |  | 1,412/8,958 | 1.05 (0.94-1.17) | 0.422 |
| Chronic bronchitis or emphysema | 935/12,725 | 1.00 (0.82-1.21)***^c^*** | 0.963 |  | 679/8,938 | 1.00 (0.86-1.16) | 0.981 |
| ***Respiratory mortality ^b^*** |  |  |  |  |  |  |  |
| Mortality from CLRD | 183/12,736 | 0.98 (0.76-1.27) | 0.895 |  | 132/8,935 | 1.18 (0.96-1.46) | 0.115 |
| Mortality from Influenza/pneumonia | 97/12,736 | 1.57 (1.24-2.00) | <0.001 |  | 81/8,935 | 0.96 (0.38-2.44)***^c^*** | 0.934 |
|  |  |  |  |  |  |  |  |
|  | Interquartile Decrease in 25-OHD | | |  | Interquartile Decrease in log_10_-Vitamin E***^d^*** | | |
|  | n/N | Effect size (95% CI) | *P*-value |  | n/N | Effect size (95% CI) | *P*-value |
| **In participants not taking supplements** |  |  |  |  |  |  |  |
| ***Respiratory morbidity ^a^*** |  |  |  |  |  |  |  |
| Current asthma | 740/13,883 | 1.05 (0.89-1.25) | 0.579 |  | 736/15,241 | 1.07 (0.90-1.29) | 0.442 |
| Wheeze in past 12 months | 1,929/13,912 | 0.97 (0.85-1.11) | 0.710 |  | 2,127/15,441 | 1.08 (0.92-1.27) | 0.329 |
| Chronic bronchitis or emphysema | 873/13,880 | 1.10 (0.94-1.29) | 0.222 |  | 965/15,407 | 1.17 (0.94-1.44) | 0.159 |
| ***Respiratory mortality ^b^*** |  |  |  |  |  |  |  |
| Mortality from CLRD | 164/13,875 | 1.21 (0.87-1.67) | 0.265 |  | 184/15,399 | 1.31 (0.79-2.17) | 0.296 |
| Mortality from Influenza/pneumonia | 95/13,875 | 1.48 (1.01-2.15) | 0.043 |  | 99/15,399 | 1.48 (0.92-2.40) | 0.106 |
| **In participants taking supplements** |  |  |  |  |  |  |  |
| ***Respiratory morbidity ^a^*** |  |  |  |  |  |  |  |
| Current asthma | 721/11,106 | 0.93 (0.83-1.04) | 0.181 |  | 715/12,503 | 1.01 (0.91-1.12) | 0.870 |
| Wheeze in past 12 months | 1,715/11,139 | 1.09 (0.95-1.22) | 0.238 |  | 1,928/12,715 | 0.99 (0.93-1.05) | 0.767 |
| Chronic bronchitis or emphysema | 833/11,108 | 0.99 (0.83-1.18) | 0.934 |  | 931/12,678 | 1.05 (0.95-1.15) | 0.367 |
| ***Respiratory mortality ^b^*** |  |  |  |  |  |  |  |
| Mortality from CLRD | 159/11,115 | 1.19 (0.67-2.13) | 0.564 |  | 181/12,689 | 0.99 (0.84-1.17) | 0.927 |
| Mortality from Influenza/pneumonia | 90/11,115 | 1.29 (0.84-1.97) | 0.249 |  | 97/12,689 | 0.95 (0.70-1.30) | 0.754 |

Abbreviations: CLRD, chronic lower respiratory diseases; n, cases; N, cases and non-cases.

^a^ Effect sizes reported as odds ratios

^b^ Effect sizes reported as hazard ratios

^c^ Significant heterogeneity found between NHANES III and continuous NHANES cohorts

^d^ Vitamin E: α-tocopherol

Models stratified by vitamin supplement use were adjusted for age, sex, race/ethnicity, income, education level, cigarette smoking and exposure to cigarette smoke, pack-years of cigarette smoking, BMI, total serum cholesterol, alcohol consumption, caloric intake, general health status, and survey cycle (continuous NHANES only). Models for serum 25-OHD were additionally adjusted for season of collection, and vitamin E models were additionally adjusted for total serum cholesterol.

**Additional file 1: Table S3**: Associations between lower serum antioxidant vitamins and respiratory morbidity and mortality by smoking status [NHANES III (1988-1994) and continuous NHANES (1999-2006)]

|  | Interquartile Decrease in log_10_-Vitamin A | | |  | Interquartile Decrease in Vitamin C | | |
| --- | --- | --- | --- | --- | --- | --- | --- |
|  | n/N | Effect size (95% CI) | *P*-value |  | n/N | Effect size (95% CI) | *P*-value |
| **In smoking participants** |  |  |  |  |  |  |  |
| ***Respiratory morbidity ^a^*** |  |  |  |  |  |  |  |
| Current asthma | 748/13,090 | 0.92 (0.82-1.04) | 0.209 |  | 613/9,862 | 1.07 (0.91-1.26) | 0.423 |
| Wheeze in past 12 months | 2,555/13,305 | 1.07 (0.94-1.22) | 0.318 |  | 1,953/9,893 | 1.09 (1.00-1.19) | 0.039 |
| Chronic bronchitis or emphysema | 1,378/13,263 | 1.04 (0.96-1.14) | 0.349 |  | 1,060/9,868 | 1.09 (0.94-1.26) | 0.272 |
| ***Respiratory mortality ^b^*** |  |  |  |  |  |  |  |
| Mortality from CLRD | 310/13,275 | 1.31 (0.88-1.94)***^c^*** | 0.180 |  | 234/9,872 | 1.26 (1.04-1.54) | 0.020 |
| Mortality from Influenza/pneumonia | 106/13,275 | 1.24 (1.05-1.45) | 0.009 |  | 92/9,872 | 0.91 (0.56-1.48) | 0.720 |
| **In non-smoking participants** |  |  |  |  |  |  |  |
| ***Respiratory morbidity ^a^*** |  |  |  |  |  |  |  |
| Current asthma | 705/14,725 | 0.92 (0.78-1.09) | 0.357 |  | 580/10,791 | 0.99 (0.83-1.20) | 0.960 |
| Wheeze in past 12 months | 1,509/14,928 | 0.98 (0.86-1.10) | 0.718 |  | 1,117/10,815 | 1.06 (0.92-1.22) | 0.449 |
| Chronic bronchitis or emphysema | 524/14,899 | 1.00 (0.86-1.16) | 0.980 |  | 389/10,793 | 0.96 (0.79-1.16) | 0.679 |
| ***Respiratory mortality ^b^*** |  |  |  |  |  |  |  |
| Mortality from CLRD | 58/14,890 | 0.84 (0.30-2.31)***^c^*** | 0.748 |  | 45/10,781 | 1.31 (0.61-2.77) | 0.487 |
| Mortality from Influenza/pneumonia | 91/14,890 | 1.15 (0.73-1.82) | 0.565 |  | 74/10,781 | 1.09 (0.58-2.08) | 0.795 |
|  |  |  |  |  |  |  |  |
|  | Interquartile Decrease in 25-OHD | | |  | Interquartile Decrease in log_10_-Vitamin E***^d^*** | | |
|  | n/N | Effect size (95% CI) | *P*-value |  | n/N | Effect size (95% CI) | *P*-value |
| **In smoking participants** |  |  |  |  |  |  |  |
| ***Respiratory morbidity ^a^*** |  |  |  |  |  |  |  |
| Current asthma | 752/11,869 | 0.99 (0.86-1.13) | 0.855 |  | 746/13,061 | 1.10 (0.96-1.28) | 0.180 |
| Wheeze in past 12 months ***^e^*** | 2,295/11,902 | 1.05 (0.92-1.20) | 0.503 |  | 2,551/13,274 | 1.11 (1.04-1.19) | 0.001 |
| Chronic bronchitis or emphysema ***^f^*** | 1,231/11,865 | 1.08 (0.94-1.23) | 0.281 |  | 1,374/13,232 | 1.13 (1.03-1.24) | 0.007 |
| ***Respiratory mortality ^b^*** |  |  |  |  |  |  |  |
| Mortality from CLRD | 270/11,876 | 1.33 (1.00-1.77) | 0.046 |  | 309/13,244 | 1.11 (0.93-1.33) | 0.267 |
| Mortality from Influenza/pneumonia ***^g^*** | 103/11,876 | 1.75 (0.96-3.18) | 0.065 |  | 106/13,244 | 0.84 (0.62-1.15) | 0.292 |
| **In non-smoking participants** |  |  |  |  |  |  |  |
| ***Respiratory morbidity ^a^*** |  |  |  |  |  |  |  |
| Current asthma | 709/13,120 | 1.01 (0.87-1.18) | 0.868 |  | 705/14,683 | 0.96 (0.84-1.09) | 0.506 |
| Wheeze in past 12 months ***^e^*** | 1,349/13,149 | 1.00 (0.86-1.15) | 0.955 |  | 1,504/14,882 | 0.91 (0.82-1.02) | 0.093 |
| Chronic bronchitis or emphysema ***^f^*** | 475/13,123 | 0.97 (0.72-1.32) | 0.871 |  | 522/14,853 | 0.94 (0.82-1.08) | 0.415 |
| ***Respiratory mortality ^b^*** |  |  |  |  |  |  |  |
| Mortality from CLRD | 53/13,114 | 0.74 (0.34-1.63) | 0.462 |  | 56/14,844 | 0.89 (0.67-1.17) | 0.399 |
| Mortality from Influenza/pneumonia ***^g^*** | 82/13,114 | 0.80 (0.50-1.28) | 0.352 |  | 90/14,844 | 1.21 (0.95-1.55) | 0.129 |

Abbreviations: CLRD, chronic lower respiratory diseases; n, cases; N, cases and non-cases.

^a^ Effect sizes reported as odds ratios

^b^ Effect sizes reported as hazard ratios

^c^ Significant heterogeneity found between NHANES III and continuous NHANES cohorts

^d^ Vitamin E: α-Tocopherol

^e^ *P*_interaction_<0.01 for Smoking*Vitamin E

^f^ *P*_interaction_<0.01 for Smoking*Vitamin E

^g^ *P*_interaction_<0.01 for Smoking*25-OHD

Models stratified by smoking status were adjusted for age, sex, race/ethnicity, income, education level, use of vitamin supplements, exposure to cigarette smoke, pack-years of cigarette smoking (among smokers only), BMI, alcohol consumption, caloric intake, general health status, and survey cycle (continuous NHANES only). Models for serum 25-OHD were additionally adjusted for season of collection, and vitamin E models were additionally adjusted for total serum cholesterol.

**Additional file 1: Table S4:** Cohort specific estimates in models with significant heterogeneity

|  | NHANES III | |  | | Continuous NHANES | | |  | |
| --- | --- | --- | --- | --- | --- | --- | --- | --- | --- |
|  | n/N | Effect size (95% CI) | *P*-value |  | n/N | Effect size (95% CI) | *P*-value | *P*_heterogeneity_ |  |
| **Interquartile Decrease in log_10_-Vitamin A** |  |  |  |  |  |  |  |  |  |
| Chronic bronchitis or emphysema in participants taking supplements | 403/5,240 | 1.11 (0.95-1.29) | 0.204 |  | 532/7,485 | 0.91 (0.81-1.02) | 0.108 | 0.046 |  |
| Mortality from CLRD in participants not taking supplements | 121/8,496 | 2.03 (1.58-2.61) | <0.001 |  | 64/6,933 | 1.19 (0.87-1.64) | 0.274 | 0.010 |  |
| Mortality from CLRD in smoking participants | 178/6,695 | 1.61 (1.21-2.15) | 0.001 |  | 132/6,580 | 1.08 (0.84-1.38) | 0.551 | 0.039 |  |
| Mortality from CLRD in non-smoking participants | 40/7,030 | 0.50 (0.27-0.92) | 0.025 |  | 18/7,860 | 1.40 (0.80-2.45) | 0.246 | 0.015 |  |
|  |  |  |  |  |  |  |  |  |  |
| **Interquartile Decrease in Vitamin C** |  |  |  |  |  |  |  |  |  |
| Mortality from influenza/pneumonia in participants taking supplements | 67/5,098 | 0.63 (0.43-0.94) | 0.025 |  | 14/3,837 | 1.66 (0.74-3.74) | 0.225 | 0.037 |  |
|  |  |  |  |  |  |  |  |  |  |
| **Vitamin C Deficiency** |  |  |  |  |  |  |  |  |  |
| Chronic bronchitis or emphysema | 197/2,158 | 0.94 (0.72-1.21) | 0.618 |  | 76/460 | 1.81 (1.35-2.43) | <0.001 | <0.001 |  |

Abbreviations: CLRD, chronic lower respiratory diseases; n, cases; N, cases and non-cases.

Models were adjusted for age, sex, race/ethnicity, income, education level, use of vitamin supplements (where appropriate), cigarette smoking and exposure to cigarette smoke, pack-years of cigarette smoking (where appropriate), BMI, alcohol consumption, caloric intake, general health status, and survey cycle (continuous NHANES only).

**Additional file 1: Table S5**: Association of vitamin C deficiency and vitamin D (25-OHD) insufficiency with respiratory morbidity and mortality [NHANES III (1988-1994) and continuous NHANES (1999-2006)]

|  |  | Vitamin C Deficiency | | |  | Vitamin D Insufficiency | | |
| --- | --- | --- | --- | --- | --- | --- | --- | --- |
|  | Population | n/N | Effect size (95% CI) | *P*-value |  | n/N | Effect size (95% CI) | *P*-value |
| ***Respiratory Morbidity ^a^*** |  |  |  |  |  |  |  |  |
| Current asthma | NHANES III | 102/2,156 | 1.04 (0.76-1.44) | 0.808 |  | 310/6,258 | 1.16 (0.91-1.47) | 0.224 |
|  | Continuous NHANES | 45/457 | 1.00 (0.64-1.57) | 0.998 |  | 321/4,198 | 1.00 (0.86-1.16) | 0.966 |
|  | Pooled | 147/2,613 | 1.03 (0.79-1.33) | 0.854 |  | 631/10,456 | 1.04 (0.91-1.20) | 0.543 |
| Wheeze in the past  12 months | NHANES III | 418/2,165 | 0.97 (0.81-1.16) | 0.717 |  | 957/6,266 | 0.99 (0.82-1.19) | 0.891 |
|  | Continuous NHANES | 111/460 | 1.16 (0.86-1.56) | 0.344 |  | 636/4,211 | 1.18 (0.99-1.41) | 0.073 |
|  | Pooled | 529/2,625 | 1.01 (0.87-1.18) | 0.874 |  | 1,593/10,477 | 1.08 (0.91-1.29) | 0.385 |
| Chronic bronchitis or  emphysema | NHANES III | 197/2,158 | 0.94 (0.72-1.21) | 0.618 |  | 393/6,257 | 0.99 (0.78-1.25) | 0.940 |
|  | Continuous NHANES | 76/460 | 1.81 (1.35-2.43) | <0.001 |  | 297/4,194 | 1.07 (0.84-1.38) | 0.587 |
|  | Pooled | 273/2,618 | 1.30 (0.68-2.47)^c^ | 0.439 |  | 690/10,451 | 1.03 (0.87-1.22) | 0.764 |
| ***Respiratory Mortality ^b^*** |  |  |  |  |  |  |  |  |
| Mortality from CLRD | NHANES III | 45/2,160 | 1.03 (0.56-1.89) | 0.919 |  | 98/6,242 | 1.54 (0.97-2.46) | 0.069 |
|  | Continuous NHANES | 4/483 | 0.68 (0.12-3.86) | 0.655 |  | 40/4,460 | 1.12 (0.74-1.69) | 0.581 |
|  | Pooled | 49/2,643 | 0.98 (0.56-1.72) | 0.960 |  | 138/10,702 | 1.29 (0.94-1.76) | 0.110 |
| Mortality from influenza  or pneumonia | NHANES III | 26/2,160 | 1.69 (0.79-3.61) | 0.170 |  | 59/6,242 | 1.79 (1.11-2.89) | 0.018 |
|  | Continuous NHANES | 4/483 | 3.39 (1.09-10.52) | 0.036 |  | 18/4,460 | 1.31 (0.52-3.29) | 0.562 |
|  | Pooled | 30/2,643 | 2.12 (1.12-4.02) | 0.021 |  | 77/10,702 | 1.67 (1.11-2.53) | 0.015 |

Abbreviations: CLRD, chronic lower respiratory diseases; n, cases; N, cases and non-cases.

^a^ Effect sizes reported as odds ratios

^b^ Effect sizes reported as hazard ratios

^c^ Significant heterogeneity found between NHANES III and continuous NHANES cohorts

Models were adjusted for age, sex, race/ethnicity, income, education level, use of vitamin supplements, cigarette smoking and exposure to cigarette smoke, pack-years of cigarette smoking, BMI, alcohol consumption, caloric intake, general health status, and survey cycle (continuous NHANES only). Vitamin D models were additionally adjusted for season of collection. Vitamin C deficiency was defined as serum levels <0.20 mg/dL and vitamin D insufficiency as serum levels <20 ng/mL. The association between vitamin D deficiency (serum levels < 12 ng/mL) and respiratory outcomes could not be estimated due to sparse data.
